# Our Contemporaries

**Published:** 1914-05

**Authors:** 


### OUR CONTEMPORARIES

The May issue of the Medical Review of Reviews will be a woman's number, all the contributed articles being from distinguished women physicians. We published such an issue in June, 1896, and are disposed to issue another.

The American Medical Journal holds that Utah leads the states because it has a law passed in 1907 prohibiting any board of health, education, etc., to compel vaccination.

Medical Freedom mentions an epidemic of smallpox in Minto in which nine members of a family who had been vaccinated, contracted the disease, the only member escaping being a girl who escaped vaccination. The Massachusetts Senate passed a bill on April 10, providing that opposition to vaccination shall not exclude a pupil from school. We would be pleased to have definite information as to the Minto epidemic, but have not been able to locate the place.

